# Evaluation of the Selected Mechanical and Aesthetic Properties of Experimental Resin Dental Composites Containing 1-phenyl-1,2 Propanedione or Phenylbis(2,4,6-trimethylbenzoyl)-phosphine Oxide as a Photoinitiator

**DOI:** 10.3390/ijms24065573

**Published:** 2023-03-14

**Authors:** Andrea Kowalska-Kuczyńska, Jerzy Sokołowski, Małgorzata Iwona Szynkowska-Jóźwik, Tomasz Gozdek, Katarzyna Klajn, Karolina Kopacz, Kinga Bociong

**Affiliations:** 1 Department of General Dentistry, Medical University of Lodz, 92-213 Lodz, Poland; jerzy.sokolowski@umed.lodz.pl; 2 Faculty of Chemistry, Institute of General and Ecological Chemistry, Lodz University of Technology, Zeromskiego 116, 90-543 Lodz, Poland; malgorzata.szynkowska@p.lodz.pl; 3 Institute of Polymer & Dye Technology, Lodz University of Technology, Stefanowskiego 12/16, 90-924 Lodz, Poland; tomasz.gozdek@p.lodz.pl (T.G.); katarzyna.klajn@p.lodz.pl (K.K.); 4 “DynamoLab” Academic Laboratory of Movement and Human Physical Performance, Medical University of Lodz, Pomorska 251, 92-215 Lodz, Poland; karolina.kopacz@umed.lodz.pl; 5 Department of Health Sciences, Medical University of Mazovia, Rydygiera 8, 01-793 Warszawa, Poland

**Keywords:** photoinitiator, camphorquinone, PPD, BAPO, dental composite, experimental composite, resin-based composite, restorative dentistry, preventive dentistry

## Abstract

The goal of this study was to compare the mechanical properties of experimental resin dental composites containing a conventional photoinitiating system (camphorquinone CQ and 2-(dimethylami-no)ethyl methacrylate (DMAEMA)) to a photoinitiator system containing 1-phenyl-1,2 propanedione (PPD) with 2-(dimethylami-no)ethyl methacrylate) or acting alone phenylbis(2,4,6-trimethylbenzoyl)-phosphine oxide (BAPO). The manually produced composites consisted of an organic matrix: bis-GMA (60 wt. %), TEGDMA (40 wt. %), and silanized silica filler (45 wt. %). The composites contained 0.4/0.8 wt. %, 0.8/1.6 wt. %, and 1/2 wt. % of PPD/DMAEMA and another group included 0.25, 0.5, or 1 wt. % of BAPO. Vickers hardness, microhardness (in the nanoindentation test), diametral tensile strength, and flexural strength were assessed, and CIE L* a* b* colorimetric analysis was conducted for each composite produced. The highest average Vickers hardness values were obtained for the composite containing 1 wt. % BAPO (43.73 ± 3.52 HV). There was no statistical difference in the results of diametral tensile strength for the experimental composites tested. The results of 3-point bending tests were the highest for composites containing CQ (77.3 ± 8.84 MPa). Despite the higher hardness of experimental composites including PPD or BAPO, compared with composites with CQ, the overall results indicate that the composite with CQ still represents a better solution when used as a photoinitiator system. Moreover, the composites containing PPD and DMAEMA are not promising in terms of color or mechanical properties, especially as they require significantly longer irradiation times.

## 1. Introduction

Dental composites have been studied for almost fifty years. Every component has been changed or improved to obtain better mechanical and biocompatible features. The one constant in the process of forming polymer chains is the polymerization process. Polymerization can be initiated by various physical media; in the case of photopolymerization, this trigger is a light. Resin-based composites consist of monomers; after exposure to light, they form a polymer [1]. Exposure to light promotes the excitation of the photoinitiator; after this, the reactive photoinitiator starts to generate free radicals [2]. The newly formed radicals break the double bonds of the monomers, and then the monomers bind together and form long chains of polymers [1]. In recent years, camphorquinone and tertiary amines have become the gold standard in photoinitiator systems and are used in most commercial dental resins. As stated in our last study [3], a composite including 0.75 wt. % of diphenyl 2,4,6-(trimethylbenzoyl) phosphine oxide (TPO) has optimal mechanical features and is less yellow in color than composites with a camphorquinone/amine photoinitiating system. This article is a continuation of our research into the mechanical features of experimental composites containing photoinitiators other than camphorquinone (CQ), and seeks to determine the optimal concentration of photoinitiator systems [3].

The most commonly used photoinitiator in dental resin materials is camphorquinone. This is an alpha-diketone, and the range of absorbance is 360–510 nm [4]; the maximum absorbance is 468 nm [4,5,6]. It takes the form of a yellow powder, which has poor bleaching properties due to the residues of unreacted molecules of CQ [7,8,9]. Camphorquinone is a Type II photoinitiator, and it requires a co-initiator as a donor of electrons and protons in its excited state to generate radicals [10]. Tertiary amines are added as a co-initiator to speed up the process of polymerization and increase the depth of cure [7,9,11,12]. This system has many advantages, including a depth of cure of more than 2 mm; moreover, it is compatible with most dental curing units. Additionally, its effect on strength, hardness, depth of cure, water sorption, and polymerization shrinkage has been tested many times and under different conditions, and this system established the standards for optimal mechanical properties. 

In this study, the features of 1-phenyl-1,2 propanedione (PPD) or phenylbis(2,4,6-trimethylbenzoyl)-phosphine oxide (BAPO) are compared to the mechanical characteristics of camphorquinone. PPD is an alpha-diketon and has two groups: on one side a carbonyl group, and a methyl group on the other side [12,13]. This photoinitiator is an eye-catching liquid; its range of absorbance is 300–400 nm, and the absorbance maximum is 410 nm [12,13,14], which is similar to the range of CQ [15]. There is evidence that PPD can be used without co-initiators, which can be advantageous. Another feature of PPD is an improvement of crosslinking by monomers in the network, which has an effect on polymerization [16]. This photoinitiator is not used in the production of commercial dental composites. 

Another potential photoinitiator used in our experimental composite is phenylbis(2,4,6-trimethylbenzoyl)-phosphine oxide (BAPO), another name for which is Irgacure 819 [17]. BAPO is similar to TPO; it is a derivative of phosphine oxide and it does not require a co-initiator [18,19]. The absorption range is 365–416 nm, and the absorbance maximum is 400 nm [20], which another source reports as 371 nm [12]. This absorption range requires the use of dental lamps which have a wider spectrum of light; this is a significant disadvantage of using this photoinitiator. BAPO is solid and has a symmetric chemical structure and poor solubility in a variety of monomers [21]. BAPO is a type-I photoinitiator that absorbs high levels of energy from violet light and undergoes cleavage of carbon-phosphorus bonds. This process is called alpha-cleavage, whereby the compound of BAPO breaks into radicals [20]. BAPO produces more free radicals than TPO because it has two carbonyl groups in its structure. Four reactive radicals can be generated from one molecule of BAPO [15,22]. Derivates of BAPO are used in the ink industry but have not been used in dentistry [23].

The premise of this paper is to compare the properties of experimental composites containing different concentrations of PPD or BAPO to the properties of CQ and tertiary amines in experimental resin dental composites. Furthermore, we aim to assess the influence of the concentration of PPD or BAPO on hardness, flexural strength, diametral tensile strength, and color. The null hypothesis is that dental resin containing BAPO or PPD performs no worse than composites with CQ/tertiary amines, in terms of its properties.

## 2. Results

a. The influence of the amounts of PPD and DMAEMA on the mechanical properties of the experimental composites

**Figure 1:**
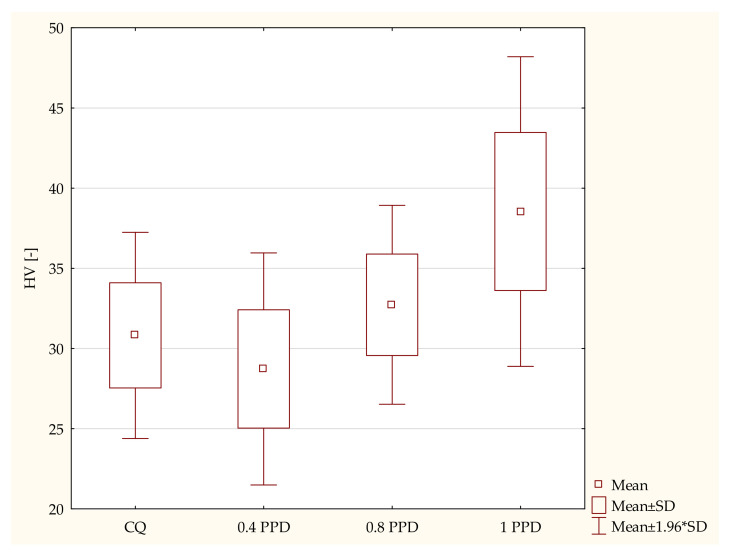
The influence of the amount of PPD (wt. %) in comparison with CQ on the hardness of a composite polymerized on both sides for 20 s using 1450 mW/cm^2^ irradiance output.

The control group containing CQ and DMAEMA had Vicker’s hardness levels that were very similar to those of the samples containing PPD and DMAEMA. The samples containing the highest concentrations of PPD and DMAEMA had the highest values of Vicker’s hardness, even higher than those of the composite with CQ as the photoinitiator. In the description below, the concentrations of DMAEMA (amounting to twice as much as the amount of CQ) are omitted to make the report more understandable. According to the ANOVA test, a statistically significant difference was demonstrated in the HV of the composites with PPD as a photoinitiator (*p*-value = 0.00000). The post hoc Scheffe test showed statistically significant differences between the following specimens (Figure 1):

- Samples containing CQ and 1 wt. % of PPD (*p*-value = 0.00044), with higher values in the composite with 1 wt. % PPD.
- The dental composite including PPD 0.4 wt. % and PPD 1 wt. % (*p*-value = 0.00000), with higher values in the composite with 1 wt. % PPD.
- Specimens containing 0.8 wt. % PPD and 1wt. % PPD (*p*-value = 0.01090), with higher values in the dental resin with 1 wt. % PPD.

To measure the hardness of the different levels inside the samples, a nanoindentation test was performed (Table 1). The measurement was made on the same length of every sample. At every length, the highest values of microhardness were achieved by composites with 1 wt. % of PPD. The higher the concentration of PPD in the dental composite, the better the microhardness results obtained. Comparing the values of PPD to values of composites with golden mean, the higher results have samples with CQ/DMAEMA. 

According to the ANOVA test, no statistically significant difference was demonstrated in terms of the diametral tensile strength of the experimental composites containing PPD (*p*-value = 0.49689). 

**Figure 2:**
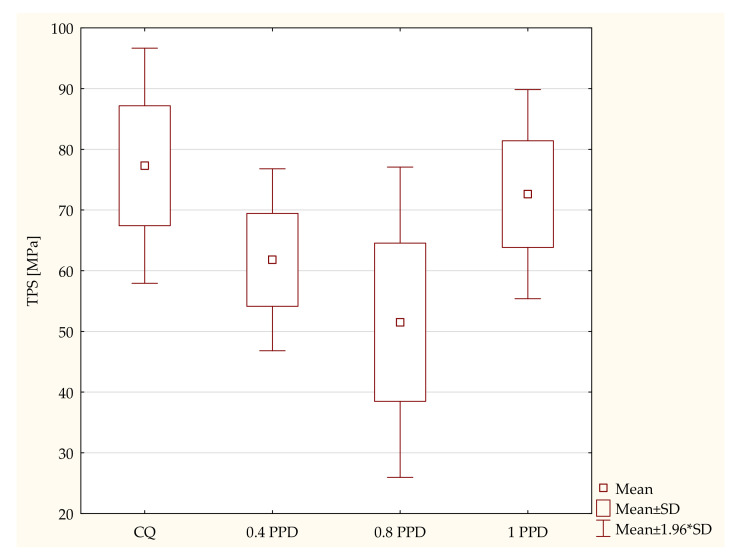
The influence of PPD concentration (wt. %) in comparison with CQ on the 3-point bending flexural strength (TPS) of composited polymerized on each side for 120 s using 1450 mW/cm^2^ irradiance output.

Next, a three-point bending test was carried out; this test determines the flexural strength and the modulus of elasticity in bending. The samples containing CQ as a photoinitiator had higher levels of flexural strength. However, the higher the concentration of PPD the composite included, the higher the values of flexural strength it obtained. According to the ANOVA test, a statistically significant difference was demonstrated in the resin-based composites containing PPD in TPS (*p*-value = 0.00123). The post hoc Scheffe test showed statistically significant differences between the following specimens (Figure 2):

- Samples containing CQ and 0.8 wt. % PPD (*p*-value = 0.00379), with higher values in composites containing CQ;
- Experimental composites with 0.8 wt. % PPD and 1 wt. % PPD (*p*-value = 0.01334), with higher values in resin containing 1 wt. % PPD.

**Figure 3:**
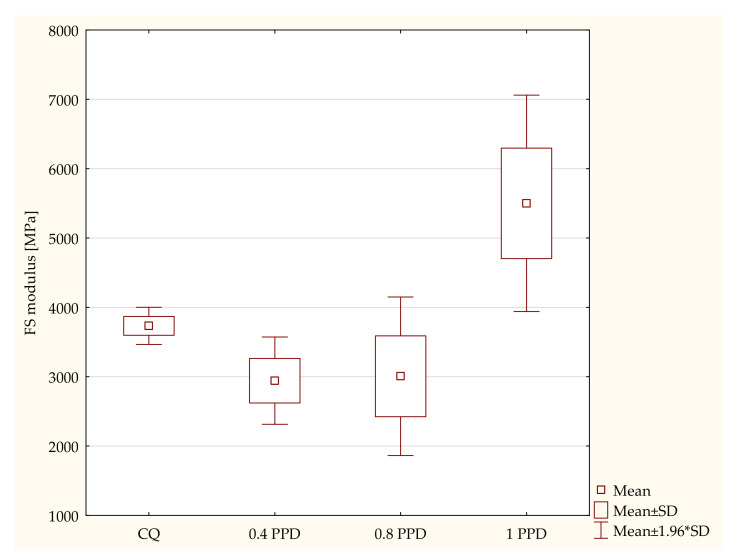
The influence of the amount of PPD (wt. %) in comparison with CQ on the modulus of elasticity in bending of composites polymerized on each side for 120 s using 1450 mW/cm^2^ irradiance output.

During the same test, the modulus of elasticity in bending was specified. The highest values of the modulus of elasticity are represented by the composite containing 1 wt. % PPD. According to the ANOVA test, a statistically significant difference was demonstrated in the composites with PPD in terms of the FS modulus (*p*-value = 0.00000). The post hoc Scheffe test showed statistically significant differences between the following specimens (Figure 3):

- Composites with CQ and 1 wt. % PPD (*p*-value = 0.00035), with higher values in dental resins with 1 wt. % PPD.
- Experimental dental resins with 0.4 wt. % and 1 wt. % PPD (*p*-value = 0.00000), with higher values in specimens with 1 wt. % PPD.
- Samples with 0.8 wt. % PPD and 1 wt. % PPD (*p*-value = 0.00000), with higher values in composites with 1 wt. % PPD.

**Figure 4:**
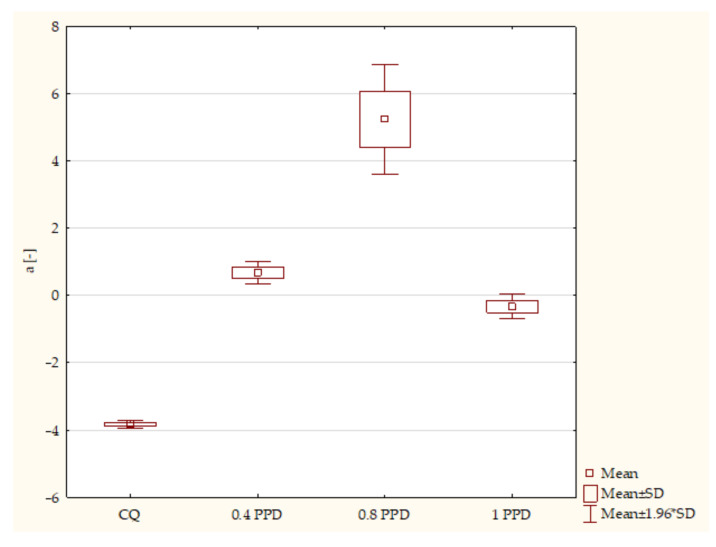
The influence of the amount of PPD (wt. %) in comparison with CQ on color measurement of CIE L* a* b* according to the axis a* of composites polymerized on each side for 20 s using 1450 mW/cm^2^ irradiance.

The results of the CIE L* a* b* color system are shown in two figures. According to the ANOVA test, a statistically significant difference was demonstrated for a* in composites with PPD (*p*-value = 0.00000). The post hoc Scheffe test showed statistically significant differences between the following specimens (Figure 4.):

- Composites with CQ and 0.4 wt. % PPD (*p*-value = 0.00001), with higher values in samples with 0.4 wt. % PPD;
- Samples containing CQ and 0.8 wt. % PPD (*p*-value = 0.00000), with higher values in composites with 0.8 wt. % PPD;
- Experimental resins containing CQ and 1 wt. % PPD (*p*-value = 0.00008), with higher values in specimens with 1 wt. % PPD;
- Specimens containing 0.4 wt. % PPD and 0.8 wt. % PPD (*p*-value = 0.00001), with higher values in dental resins with 0.8 wt. % PPD;
- Composites with 0.8 wt. % PPD and 1 wt. % PPD (*p*-value = 0.00000), with higher values in samples with 0.8 wt. % PPD.

**Figure 5:**
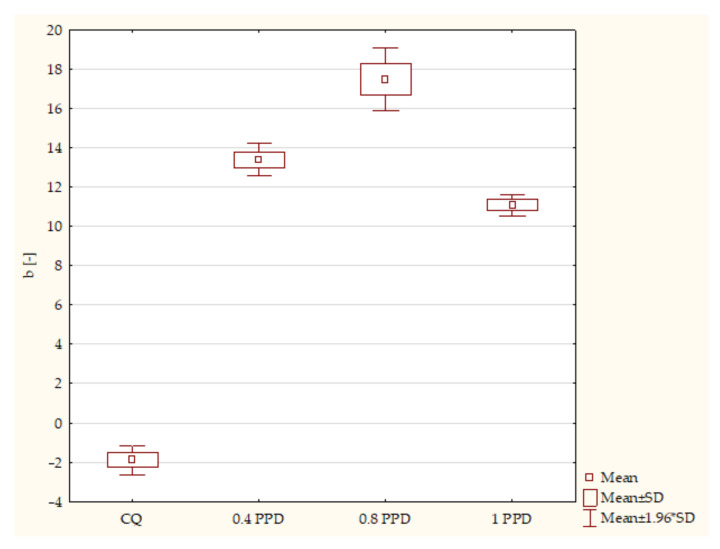
The influence of the amount of PPD (wt. %) in comparison with CQ on the color measurements of CIE L* a* b* according to the axis b* of composites polymerized on each side for 20 s using 1450 mW/cm^2^ irradiance.

According to the ANOVA test, a statistically significant difference was demonstrated for the axis b* in experimental composites with PPD (*p*-value = 0.00000). The post hoc Scheffe test showed statistically significant differences between the following samples (Figure 5):

- Composites with CQ and 0.4 wt. % PPD (*p*-value = 0.00000), with higher values in samples containing 0.4 wt. % PPD;
- Simples containing CQ and 0.8 wt. % PPD (*p*-value = 0.00000), with higher values in dental resins with 0.8 wt. % PPD;
- Experimental dental resin containing CQ and 1 wt. % PPD (*p*-value = 0.00000), with higher values in specimens with 1 wt. % PPD;
- Specimens with 0.4 wt. % PPD and 0.8 wt. % PPD (*p*-value = 0.00008), with higher values in dental resins with 0.8 wt. % PPD;
- Composites with 0.4 wt. % PPD and 1 wt. % PPD (*p*-value = 0.00445), with higher values in samples with 0.4 wt. % PPD;
- Dental resins 0.8 wt. % PPD and 1 wt. % PPD (*p*-value = 0.00000), with higher values in samples with 0.8 wt. % PPD.

b. The influence of the amount of BAPO on the mechanical properties of the experimental composites

**Figure 6:**
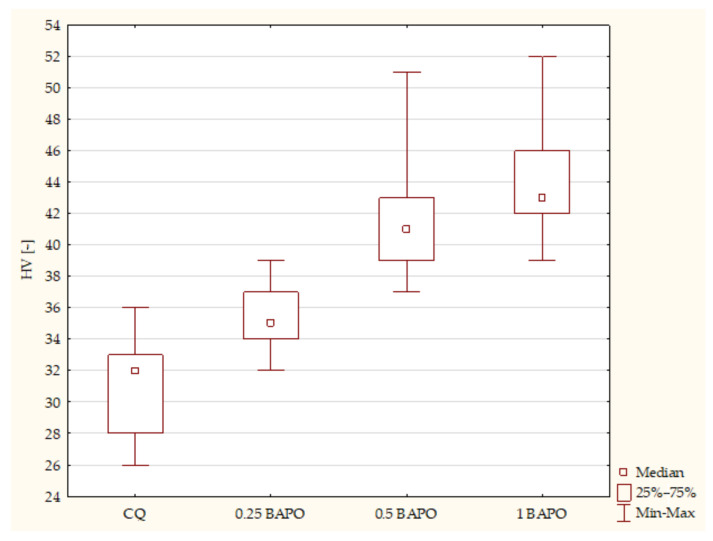
The influence of the amount of BAPO (wt. %) in comparison with CQ on the hardness of composites polymerized on both sides for 20 s using 1450 mW/cm^2^ irradiance output.

The control group samples containing CQ and DMAEMA had the lowest Vicker’s hardness values compared to samples with different concentrations of BAPO. The samples containing the highest concentration of BAPO had the highest hardness values. According to the Kruskal–Wallis test, a statistically significant difference was demonstrated in the composites with BAPO in terms of HV (*p*-value = 0.0000). The post hoc multiple comparison test showed statistically significant differences between the following samples (Figure 6):

- Composites with CQ and 0.5 wt. % BAPO (*p*-value = 0.00001), with higher values in samples with 0.5 wt. % BAPO;
- Experimental dental resins with CQ and 1 wt. % BAPO (*p*-value = 0.00000), with higher values in the specimens with 1 wt. % BAPO;
- Samples with 0.25 wt. % BAPO and 1 wt. % BAPO (*p*-value = 0.00183), with higher values in composites with 1 wt. % BAPO.

To assess the hardness inside the samples, a nanoindentation examination was conducted (Table 2). The lowest values at every level were presented by samples with 0.25 wt. % of BAPO. The highest values of microhardness were achieved by samples with BAPO 0.5 wt. % at 900 µm; however, at deeper layers, this value was significantly lower. This tendency is also noticeable in samples with 1 wt. % of BAPO. However, at lengths of 1350 µm, the highest values of microhardness were achieved by the composite containing CQ/DMAEMA. 

It is also worth noting that, according to the ANOVA test, no statistically significant difference was found in the composites with BAPO in terms of DTS (*p*-value = 0.12394).

**Figure 7:**
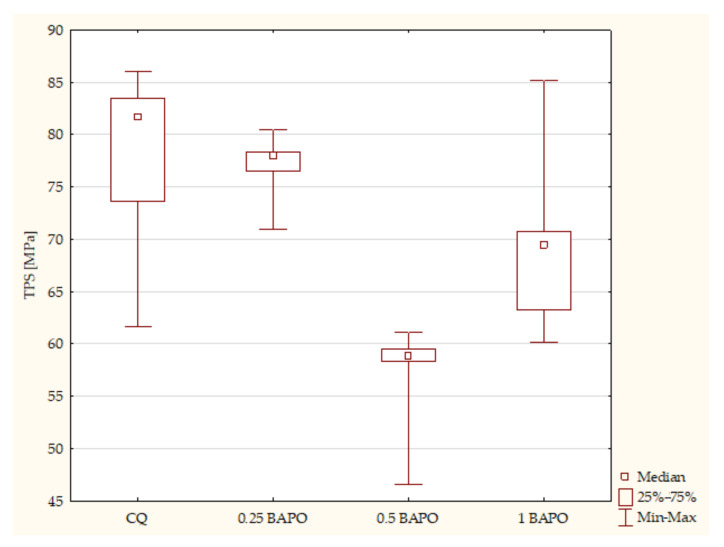
The influence of the amount of BAPO (wt. %) in comparison with CQ on the three-point bending flexural strength (FS) of composites polymerized on both sides for 20 s using 1450 mW/cm^2^ irradiance output.

The samples containing CQ as a photoinitiator had higher values of flexural strength than specimens with different concentrations of BAPO. However, the composites with the lowest concentration of BAPO had the highest value of TPS among the experimental resins containing BAPO. According to the Kruskal–Wallis test, a statistically significant difference was demonstrated in the samples containing BAPO in terms of TPS (*p*-value = 0.0035). The post hoc multiple comparison test showed statistically significant differences between the following samples (Figure 7):

- Composites with CQ and 0.5 wt. % BAPO (*p*-value = 0.01161), with higher values in the control group;
- Experimental resins with 0.25 wt. % BAPO and 1 wt. % BAPO (*p*-value = 0.02761), with higher values in composites with 0.25 wt. % BAPO.

According to the ANOVA test, no statistically significant difference was demonstrated in the BAPO in the FS modulus (*p*-value = 0.56756).

**Figure 8:**
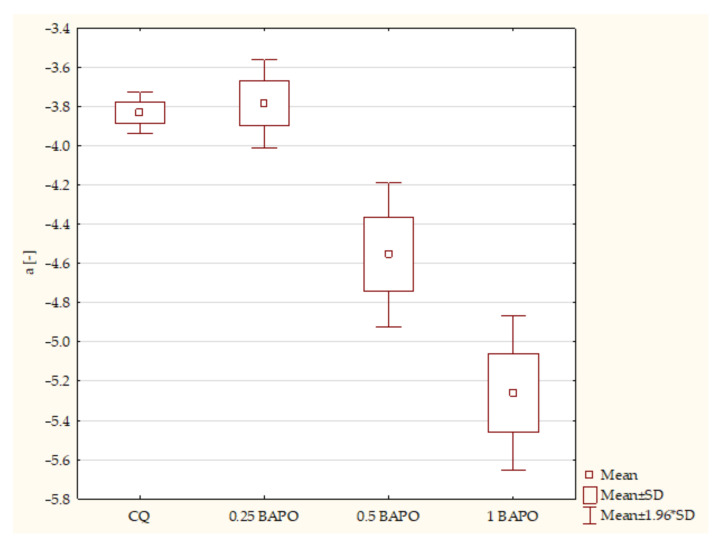
The influence of the amount of BAPO (wt. %) in comparison with CQ on the color measurement of CIE L* a* b* according to the a* axis of composites polymerized on both sides for 20 s using 1450 mW/cm^2^ irradiance output.

The results of experimental composites containing different concentrations of BAPO and of the control group concerning axis a* are stated below. According to the ANOVA test, a statistically significant difference was demonstrated in the a* axis in composites with BAPO (*p*-value = 0.00000). The post hoc Scheffe test showed statistically significant differences between the following samples (Figure 8):

- Composites with CQ and 0.5 wt. % BAPO (*p*-value = 0.00151), with higher values in samples containing CQ;
- Dental resins containing CQ and 1 wt. % BAPO (*p*-value = 0.00003), with higher values in specimens with CQ;
- Experimental composites with 0.25 wt. % BAPO and 0.5 wt. % BAPO (*p*-value = 0.00096), with higher values in composites with 0.25 wt. % BAPO;
- Specimens containing 0.25 wt. % BAPO and 1 wt. % BAPO (*p*-value = 0.00001), with higher values in samples with 0.25 wt. % BAPO;
- Samples containing 0.5 wt. % BAPO and 1 wt. % BAPO (*p*-value = 0.00184), with higher values in composites with 0.5 wt. % BAPO.

**Figure 9:**
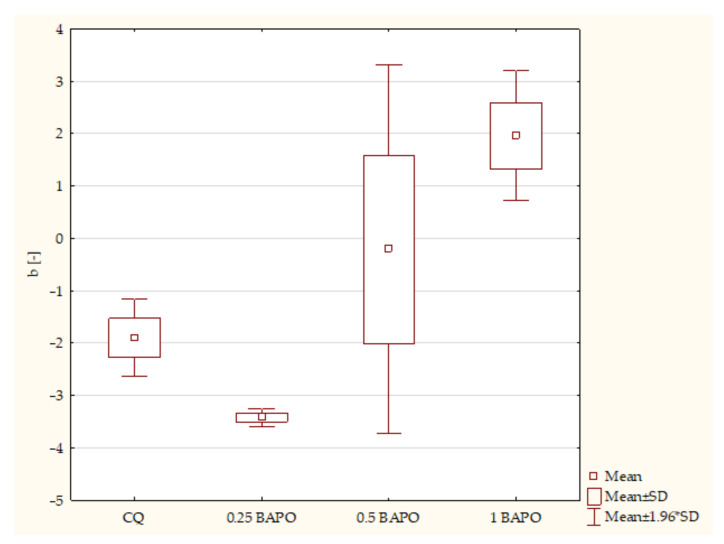
The influence of the amount of BAPO (wt. %) in comparison with CQ on the color measurement of CIE L* a* b* according to axis b* of composites polymerized on both sides for 20 s using 1450 mW/cm^2^ irradiance output.

According to the ANOVA test, a statistically significant difference was demonstrated in the b* axis in composites containing BAPO (*p*-value = 0.001128). The post hoc Scheffe test showed statistically significant differences between the following samples (Figure 9):

- Composites with CQ and 1 wt. % BAPO (*p*-value = 0.01428), with higher values in samples with 1 wt. % BAPO;
- Dental resins containing 0.25 wt. % BAPO and 0.5 wt. % BAPO (*p*-value = 0.02739), with higher values in specimens with 0.5 wt. % BAPO;
- Dental resins with 0.25 wt. % BAPO and 1 wt. % BAPO (*p*-value = 0.00167), with higher values in composites with 1 wt. % BAPO.

## 3. Discussion

This article compares the different characteristics of dental resin composites containing CQ/DMAEMA and various concentrations of PPD with DMAEMA or BAPO as photoinitiators. Analysis of this range highlights the most important mechanical properties of experimental dental composites. The null hypothesis of our study is that composites containing PPD and DMAEMA or BAPO have properties that are no worse than those of dental resins produced according to the gold standard.

The first test we conducted was the Vicker’s hardness test, which we used in place of the more commonly used degree of conversion of dental composites. This method was chosen because overestimations are possible in relation to the degree of conversion, and Knoop or Vicker’s hardness is easy to assess, more accurate, and represents a property that is more clinically useful [24,25]. The statistics showed that composites containing PPD and DMAEMA have lower values of hardness than composites containing CQ and DMAEMA. Only the dental resins containing 1 wt. % of PPD and 2 wt. % DMAEMA have a higher Vicker’s hardness. The degree of conversion is often used as an indirect measure of hardness and an indicator of cross-linking [26]. In their study, Park et al. [27] compared the degree of conversion of different concentrations of CQ and PPD. The experimental composites containing different concentrations of PPD and N,N-cyanoethylmethylaniline (CEMA) as the reducing agent (0.2 wt. %) only had a better degree of conversion values than dental resins with CQ and the same reducing agent when the time of light exposure was higher than 200 s or when the concentration of photoinitiator was higher than 2.5 wt. % [27]. Our study considers only one curing duration (20 s, which represents the average duration of light exposure in clinical conditions), and the highest concentration of PPD was 2 wt. %. Considering the same concentration of the photoinitiator and a curing duration of 20 s, our results are consistent with the work of Park at al. [27]. Elsewhere, Brandt et al. [28] tested composites with 0.4 wt. % PPD and 0.4 wt. % CQ (0.8 wt. % DMAEMA was added to both composites) in terms of the degree of conversion and Knoop hardness after irradiation with different dental lamps. The time of irradiation was 40 s. The samples containing PPD had the lowest values of Knoop hardness regardless of the lamp that was used to cure the sample. The degree of conversion was also lower for composite with PPD and DMAEMA. Our research results are consistent with the aforementioned findings, even though different dental lamps were used in our study and the irradiation duration was longer [28]. Another study by Brandt et al. was performed in 2010; it compared the degree of conversion of PPD 0.8 wt. % and CQ 0.8 wt. %. In this study, samples with PPD achieved a lower degree of conversion even when different lamps were used [29]. Resende et al. [30] examined the degree of conversion and Knoop hardness of experimental composites containing PPD, BAPO, and CQ. The irradiation duration was 20 s, and the filler content was 65%. The values of Knoop hardness and the degree of conversion were the lowest for composites containing 0.2 wt. % of PPD and 0.8 wt. % of DMAEMA [30].

The nanoindentation values of composites containing different concentrations of PPD and DMAEMA relative to the control group confirm that CQ/DMAEMA represents the superior photoinitiator system. It is worth noting that the composite samples containing PPD were shorter; their height was only 1 mm, whereas the samples with BAPO or CQ were 2 mm high. When the samples with PPD were taller than 1mm, the bottom of the samples was still uncured after 20 s of curing. For this reason, the height was altered. The best values for microhardness were achieved by the composite with 1 wt. % of PPD in all layers. However, this sample also contains 2 wt. % DMAEMA, which causes symptoms of yellowing as its time in the oral cavity progresses [14]. 

It is noteworthy that composites containing a photoinitiator based on phosphine oxide had higher hardness values; even the lowest concentration of BAPO achieves higher values of hardness than CQ/DMAEMA. Ikemura et al. [31] showed that BAPO has a higher degree of conversion values than the resins containing CQ/EDAB. They tested unfilled resins with 2 wt. % BAPO and CQ/EDAB (0.5 wt. %/1 wt. %); in this study, the time of irradiation was 30 s. Higher values for the degree of conversion were obtained by BAPO despite different compositions of the matrix [20]. Almeida et al. also examined the degree of conversion of resins containing different photoinitiators. The irradiation time was 20 s and the concentration of BAPO was 1 mol% and CQ/EDAB (0.4 and 0.8 mol%). The resin containing BAPO achieved a degree of conversion higher than that of the resin with CQ/EDAB. Moreover, resins with BAPO achieved a higher degree of conversion than resins with TPO [31]. Such findings concur with the results of our study. Favarao et al. [32] tested resin-based cements containing different types of photoinitiator. The concentration of BAPO was 0.5 wt. % and that of CQ/EDMAB was 0.2 wt. %/0.2 wt. %. They assessed the degree of conversion of specimens, which were irradiated through veneers of different thicknesses (0.4, 0.7, 1, and 1.5 mm). For all samples containing BAPO as a photoinitiator, the degree was higher [32]. These results also support the conclusions of our research. Resende et al. [31] tested the degree of conversion and Knoop hardness of experimental composites containing PPD, BAPO, and CQ. Composites containing 0.5 wt. % BAPO had better values of Knoop hardness and the degree of conversion [31]. The superior Vicker’s hardness values and degree of conversion scores of composites containing BAPO in different concentrations are caused by the reactivity of BAPO. The greater reactivity of this photoinitiator is a result of the production of more radicals that are capable of photopolymerization; as such, this process is faster and more efficient [33,34]. 

The microhardness values of the experimental composites containing no lower amount than 0.5 wt. % BAPO show higher values on the top, inside, and at the bottom of samples. However, composites with BAPO has lower values of hardness when the distance is higher than 1 mm than samples with CQ/DMAEMA. Rocha et al. [31] noted that composites containing a photoinitiator based on derivates of phosphine oxide have better values of hardness on the top of the sample; however, at deeper levels, the values are lower. The lower levels of transmittance impair deep polymerization, despite the higher reactivity of these photoinitiators [35]. The lowest concentration of BAPO (0.25 wt. %) produces lower values of microhardness, but there was no significant difference between 0.5 wt. % and 1 wt. % of BAPO. 

The diametral tensile strength test is important when assessing a material’s deformation ability when the force acts perpendicular to the object. This test replicates the forces acting on anterior teeth and fillings of cavities of the third and fourth Black’s classes [36]. Based on the DTS results, no significant statistical difference was found; it follows that, in terms of DTS, the results for composites containing PPD and BAPO were no worse than those of the control group with CQ. This result also means that fillings with the experimental photoinitiator placed in cavities of third and fourth Black’s classes could be as durable as those with CQ in terms of tension. 

To assess flexural strength, a three-point bending test was conducted. The composites containing 1 wt. % of PPD exhibited higher values of flexural strength than resins with CQ/DMAEMA. The composites with lower concentrations of PPD demonstrated lower flexural strength than the control group. Similar dependence can be found in other studies [30]. This finding can probably be attributed to insufficient polymerization times or the use of concentrations of PPD that were too low. In our study, only the highest concentration of PPD obtained better values of flexural strength than the control group. It is possible that extending the polymerization time could improve this result. However, extending the time to more than 20 s would result in longer work times in clinical settings. 

The comparison of the results of the three-point bending test shows that the gold standard has better flexural strength than composites with BAPO. The highest flexural strength among the experimental composites with BAPO was obtained by the dental resin with 0.25 wt. % BAPO. The higher the concentration of BAPO, the lower the values of flexural strength. This tendency was also evidenced by Alves et al. [9] in their assessment of the flexural strength of resins with different concentrations of BAPO and CQ. They proved that the higher the concentration of BAPO, the lower the flexural strength. The time of curing in their study was also 20 s, and the BAPO concentration ranged from 0.2 to 2.2 wt. %. The differences between the results of Alves et al. and those obtained in our study may have been caused by the filler of the composites. In the experimental composites used by Alves et al., the filler content was 80 wt. %, whereas our composites had only 45 wt. % of filler [9]. Flexural strength also was tested by Favarao et al. [32]. They tested the samples through veneers of different thicknesses after irradiation. The flexural strength of cements with BAPO had the best values, compared to cements with CQ or TPO [32].

The final test is the CIE L* a* b* test. This method is used to determine the color tone of dental materials, such as composites and ceramics, and to compare them to the color of the dentin and enamel of natural teeth. Axis a* refers to green and red. The control group CQ/DMAEMA was the reference point. The composite containing 0.8 wt. % PPD and 1.6 wt. % DMAEMA had the warmest color. The samples with BAPO are bluer, which can cause gray and blue tints on the material filling, which are not aesthetically pleasing when compared to the natural shades of teeth. The higher the concentration of BAPO in the composite, the grayer the samples. The axis b* refers to yellow and blue. The composites with PPD as a photoinitiator are much yellower than the control group. This is caused by the color of the photoinitiator, which is an eye-catching yellow liquid. Composites containing BAPO are more yellow than specimens with CQ/DMAEMA; the higher the concentration of BAPO, the yellower the shade of the material. However, this color is not as yellow as that of the samples containing PPD/DMAEMA. The results of the CIE L* a* b* analysis of experimental composites with different concentrations of BAPO are more satisfactory. The CIE L* a* b* test was also conducted by Salgado et al. [33]. They tested experimental composites containing 1 mol% of BAPO and a control group with 1 mol% CQ and 1 mol% EDMAB; they used 60 wt. % filler and the time of irradiance was 40 s with a 600 mW/cm^2^ lamp. The results of axis a* are mostly the same for composites with BAPO and CQ. However, the values of axis b* were higher for experimental composites with BAPO. In this study, composites with BAPO are also more yellow than those with CQ [33]. Changing CQ to BAPO does not change the color of the composites or their aesthetic qualities. 

This study has some limitations. First, the samples are not the same shape as fillings in tooth cavities. The other, more important problem is that these composites have not yet been bonded to tooth tissues. There is a risk that commercially available bonding systems will not match the composites due to the different types of photoinitiators used. Despite the wide range of analyses conducted, this should be considered a pilot study that will be continued and expanded in many fields as a cycle of articles. The next steps will be the measurement of contraction stress (photoelastic analysis) generated during photopolymerization, an examination of temperature during the polymerization process, and an analysis of the genotoxicity/cytotoxicity of experimental composites with the selected types and amounts of photoinitiator. Additionally, we propose that the combination of different photoinitiators with CQ should be considered. 

## 4. Materials and Methods

The experimental dental resins used in this study are listed in Table 3. The control group is a composite containing a CQ/tertiary amine, the gold standard of modern composites. The tertiary amine 2-(dimethylamino)ethyl methacrylate (DMAEMA) (Sigma-Aldrich Inc., St. Louis, MO, USA) was used as a co-initiator of CQ. The matrix of the experimental composites was composed of 60 wt. %. bisphenol A glycerolate dimethacrylate (Bis-GMA) (Sigma-Aldrich Inc., St. Louis, MO, USA), 40 wt. % triethylene glycol dimethacrylate (TEGDMA) (Sigma-Aldrich Inc., St. Louis, MO, USA), and 0,1 wt. % 2,6-Di-tert-butyl-4-methylphenol (BHT) (Sigma-Aldrich Inc., St. Louis, MO, USA) as an inhibitor of polymerization. The composite was filled with 45 wt. % silica according to the total weight of Bis-GMA and TEGDMA (Arsil, Zakłady Chemiczne“RUDNIKI” S.A., Rudniki, Poland). Before use, the silica was silanized with γ-methacryloxypropyltrimethoxysilane (Unisil Sp. z o. o., Tarnów, Poland). The dental resins were mixed by hand in a room with no daylight or artificial light until a smooth paste was obtained. All samples were cured using a polywave Valo Lamp (Ultradent Products Inc., South Jordan, UT, USA) with three irradiance outputs (1000 mW/cm^2^, 1450 mW/cm^2^, and 3200 mW/cm^2^) and a light range of 395–510 nm. The optimal duration of curing was 20 s per 2 mm of material height. This duration was chosen to facilitate comparison with the qualities of similar experimental composites, which were explored in our recent article.

A detailed description of the performed tests can be found in our recent articles [3,37]. The specimens used for hardness and diametral tensile strength tests were cylindrical (3 mm and 6 mm diameter). They were placed in silicon molds and irradiated on both sides. As a first control, the Vickers hardness (HV) test was performed. This was measured using a semiautomatic hardness tester (ZHV2 m Zwick/Röell, Ulm, Germany). Eleven imprints were made for every experimental composite. 

The microhardness of the composites was tested using a NanoTest 600 (Micromaterials Ltd., Wrexham, Great Britain) with a Berkovich indenter. The microhardness and reduced modulus of elasticity of the composites were calculated based on no-load curves according to the method proposed by Olivier and Pharr [38]. The depths for composites containing different concentrations of PPD were 0 µm, 250 µm, 500 µm, 750 µm, and 1000 µm. The distances between measurements were 0, 450µm, 900 µm, 1350 µm, and 1800 µm for composites containing BAPO and for the control group. The maximum force was 10 mN, and the loading and unloading speed was dP/dt = 0.5 mN/s. Three measurements were made for every layer of the experimental composites.

The diametral tensile strength test (DTS) assesses the maximum resistance to a load that tends to break the specimens. The crosshead speed was 2 mm/min. The tests were performed on a universal testing machine (Z020, Zwick/Röell, Ulm, Germany). For every experimental composite, 11 samples were tested.

Flexural strength was tested using a 3-point bending test acc. PN-EN ISO 4049:2003 [39]. The specimens used in this test were rectangular (25 mm × 2 mm × 2 mm), and they were irradiated at three points twice for 20 s on each side for 120 s. The test was performed on a universal testing machine (Zwick Z020, Zwick/Röell, Ulm, Germany), with a crosshead speed of 1 mm/min. For each specimen, the maximum force required to break the samples was measured. Five measurements were taken for each experimental composite.

Finally, the colors were examined using a KONICA MINOLTA CM-3600A (Germany) spectrophotometer according to the CIE L*a*b* color system. Using this equipment, all colors can be evaluated in terms of hue, brightness, and saturation. Tests were carried out on cylindrical samples (2 mm high and 10 mm in diameter), which were cured before the test. The spectrophotometer was calibrated according to the manufacturers’ recommendations. The control group was a sample containing CQ and DMAEMA as a photoinitiator system. The CIE L*a*b* system contains three axes: a* and b* are at right angles to each other and they define the basic colors. The third axis L* refers to lightness and it is perpendicular to the plane created by the a* and b* axes. The range of axis a* is from −120 (green) to +120 (red). The scale of axis b* is from −120 (blue) to +120 (yellow). The scale of axis L* is from 0 (black saturation) to 100 (white saturation). The CIE L*a*b* system assumes that color differences are accounted for in the distance between points in the spatial arrangement of the three axes. 

Microsoft Excel from the Microsoft Office 2010 suite and Statistica v.13 were used for statistical analysis of the results. The Shapiro–Wilk normality test was used to evaluate the distribution of some parameters. If the distribution did not conform to a normal distribution, the Kruskall–Wallis test was used. In the case of a normal distribution for a particular parameter, the equality of variances was assessed using a Levene test. If the variances were equal, ANOVA with the Scheffe post hoc test was used. The accepted level of significance was α = 0.05. 

## 5. Conclusions

The mechanical properties of the composites were examined, and the color of samples was also assessed. The composites containing BAPO exhibited the highest values of hardness; moreover, the higher the concentration, the higher the hardness values. The deeper layers (greater than 1 mm) of composites with BAPO had lower values of microhardness than composites with CQ. The type and concentration of photoinitiator had an imperceptible influence on the values of diametral tensile strength. The results of three-point bending tests were better for composites containing CQ. The composites containing different concentrations of PPD and DMAEMA are poor alternatives to photoinitiator systems in terms of color, mechanical properties, and exposure time requirements. The composite containing CQ and DMAEMA has optimal mechanical features, but the addition of BAPO to the CQ could improve these properties further and reduce the number of tertiary amines. 

## Figures and Tables

**Table 1:** The influence of the amount of PPD on the average results of microhardness (MPa) and reduced modulus (GPa) with standard deviations. The results were determined using the nanoindentation method of the top (0 μm), bottom (1000 μm), and cross-section (250, 500, and 750 μm) of the tested material samples. Photopolymerization of one side 20 s per 2 mm with Valo lamp light intensity 1450 mW/cm^2^.

			PPD Amount [wt. %]			
Distance (µm)	0.4		0.8		1	
	Microhardness (MPa)	Reduced Modulus (GPa)	Microhardness (MPa)	Reduced Modulus (GPa)	Microhardness (MPa)	Reduced Modulus (GPa)
0	117.1 ± 10.1	2.5 ± 0.08	44.5 ± 4.1	1.44 ± 0.14	481.8 ± 18.1	6.27 ± 0.04
250	167.3 ± 14.1	2.9 ±0.11	234.6 ± 15.1	3.46 ± 0.06	563.6 ± 18.8	6.77 ± 0.07
500	186.3 ± 31.4	3.87 ± 0.56	247.3 ± 36.6	2.5 ± 0.23	874.5 ± 29.1	11.44 ± 0.12
750	76.4 ± 8.9	2.03 ± 0.12	203.0 ± 25.4	2.42 ± 0.34	699.9 ± 21.2	8.73 ± 0.09
1000	22.7 ± 2.3	0.86 ± 0.02	10.1 ± 3.0	0.46 ± 0.12	406.7 ± 20.0	5.77 ± 0.5

**Table 2:** The average results of microhardness (MPa) and reduced modulus (GPa) with standard deviations. The results were determined using the nanoindentation method of the top (0 μm), bottom (1800 μm), and cross-section (450, 900, and 1350 μm) of the tested material samples; photopolymerization on one side, 20 sec. per 2 mm, with a Valo lamp light intensity of 1450 mW/cm^2^.

			BAPO Amount [wt. %]					
Distance (µm)	CQ/DMAEMA		0.25		0.5		1	
	Microhardness (MPa)	Reduced Modulus (GPa)	Microhardness (MPa)	Reduced Modulus (GPa)	Microhardness (MPa)	Reduced Modulus (GPa)	Microhardness (MPa)	Reduced Modulus (GPa)
0	67.52 ± 2.48	2.15 ± 0.05	35.74 ± 5.72	1.71 ± 0.14	236.93 ± 15.97	4.77 ± 0.19	573.38 ± 19.33	7.18 ±0.18
450	352.5 ± 55.43	5.38 ±0.38	39.9 ± 8.81	1.8 ± 0.13	605.37 ± 91.38	6.66 ± 0.69	615.18 ± 33.66	6.16 ± 0.31
900	671.43 ± 89.87	5.51 ±0.1	72.61 ± 11.07	3.31 ± 0.97	793.9 ± 62.65	8.6 ±0.72	674.62 ± 141.5	6.29 ± 0.16
1350	753.49 ± 2.86	8.34 ± 0.56	87.56 ± 8.07	2.74 ± 0.09	553.93 ± 0.98	7.42 ± 1.25	533.11 ± 128.7	7.76 ± 0.74
1800	12.2 ± 1.48	0.72 ± 0.02	71.2 ± 14.01	2.39 ± 0.19	432.59 ± 32.44	5.78 ± 0.15	482.64 ± 11.92	5.76 ± 0.11

**Table 3:** The composition of the experimental composites tested in this study; 45 wt. % of silanized silica filler and 55 wt. % of a matrix containing Bis-GMA and TEGDMA.

Group	Photoinitiator System	Manufacturer	Concentration of Photoinitiator
A.	CQ and DMAEMA	Sigma-Aldrich Inc., St. Louis, MO, USA	0.4 wt. % and 0.8 wt. %
B.	BAPO		0.25 wt. %
C.	BAPO		0.5 wt. %
D.	BAPO		1 wt. %
E.	PPD and DMAEMA		0.4 wt. % and 0.8 wt. %
F.	PPD and DMAEMA		0.8 wt. % and 1.6 wt. %
G.	PPD and DMAEMA		1 wt. % and 2 wt. %

## Data Availability

The data presented in this study are available on request from the corresponding authors.

## References

[1] Al Sunbul H., Silikas N., Watts D.C. (2016). Polymerization shrinkage kinetics and shrinkage-stress in dental resin-composites. Dent. Mater..

[2] Camargo F.M., Della Bona Á., Moraes R.R., Coutinho de Souza C.R., Schneider L.F. (2015). Influence of viscosity and amine content on C==C conversion and color stability of experimental composites. Dent. Mater..

[3] Kowalska A., Sokołowski J., Szynkowska-Jó’zwik M.I., Gozdek T., Kopacz K., Bociong K. (2022). Can TPO as Photoinitiator Replace “Golden Mean” Camphorquinone and Tertiary Amines in Dental Composites? Testing Experimental Composites Containing Different Concentration of Diphenyl(2,4,6-trimethylbenzoyl)phosphine Oxide. Int. J. Mol. Sci..

[4] Van Landuyt K.L., Snauwaert J., De Muncka J., Peumansa M., Yoshidac Y., Poitevin A., Coutinho E., Suzuki K., Lambrechts P., Van Meerbeeka B. (2007). Systematic review of the chemical composition of contemporary dental adhesives. Biomaterials.

[5] Hadis M.A., Shortall A.C., Palin W.M. (2012). Competitive light absorbers in photoactive dental resin-based materials. Dent. Mater..

[6] Tay F.R., King N.M., Suh B.I., Pashley D.H. (2001). Effect of delayed activation of light-cured resin composites on bonding of all-in-one adhesives. J. Adhes Dent..

[7] Alvim H.H., Alecio A.C., Vasconcellos W.A., Furlan M., de Oliveira J.E., Saad J.R.C. (2006). Analysis of camphorquinone in com-positeresins as a function of shade. Dent. Mater..

[8] Georg H., Canuto S., Coutinho K. (2007). Solvent effects on the UV-visible absorption spectrum of benzophenone in water: A com-bined Monte Carlo quantum mechanics study including solute polarization. J. Chem. Phys..

[9] da Silva Alves Maciel D., Caires-Filho A.B., Fernandez-Garcia M., Anauate-Netto C., Alonso R.C.B. (2018). Effect of Camphorqui-none Concentration in Physical-Mechanical Properties of Experimental Flowable Resin Composites. BioMed. Res. Int..

[10] Allen N.S. (1996). Photoinitiatorsfor UV and visible curing of coatings: Mechanism and properties. Jphotochem. Photobiol. A Chem..

[11] Taira M., Urabe H., Hirose T., Wakasa K., Yamaki M. (1987). Analysis of Photo-initiators in Visible-light-cured Dental CompositeRe-sins. J. Dent. Res..

[12] Pratap B., Kant R., Bhardwaj B., Nag M. (2019). Resin based restorative dental materials: Characteristics and future perspectives. Jpn. Dent. Sci. Rev..

[13] Rueggeberg F.A., Gianninic M., Arrais C.A.G., Price R.B.T. (2017). Light curing in dentistry and clinical implications: A literature review Polymerization. Dent. Mater..

[14] Kowalska A., Sokolowski J., Bociong K. (2021). The Photoinitiators Used in Resin Based Dental Composite—A Review and Future Perspectives. Polymers.

[15] Arikawa H., Takahashi H., Kanie T., Ban S. (2009). Effect of various visible light photoinitiators on the polymerization and coloroflight-activated resins. Dent. Mater. J..

[16] Verzola K.C., Dressano D., Saraceni C.H.C., Goncalves L.S., Hadis M., Watts D.C., Palin W.M., Fonseca Lima A. (2020). Bis(4-methyl phenyl)iodonium as an alternative component to diphenyliodonium in camphorquinone-based ternary initiating systems. Dent. Mater..

[17] Neumann M.G., Miranda W.G., Schmitt C.C., Rueggeberg F.A., Correa I.C. (2005). Molar extinction coefficients and the photon absorption efficiency of dental photoinitiators and light curing units. J. Dent..

[18] Fonseca A., Salvador M.V.O., Dressano D., Saracenia C.H.C., Gonçalvesc L.S., Hadisd M., Palin W.M. (2019). Increased rates of photopolymerisation by ternary type II photoinitiator systems in dental resins. J. Mech. Behav. Biomed. Mater..

[19] Dressano D., Palialol A.R., Xavier T.A., Braga R.R., Oxman J.D., Watts D.C., Marchi G.M., Fonseca Lima A. (2016). Effect of diphenyliodonium hexafluorophosphate on the physical and chemical properties of ethanolic solvated resins containing camphorquinone and 1-phenyl-1,2-propanedione sensitizers as initiators. Dent. Mater..

[20] Ikemura K., Endo T. (2010). A review of the development of radical photopolymerization initiators used for designing light-curing dental adhesives and resin composites. Dent. Mat. J..

[21] Ullrich G., Ganster B., Salz U., Moszner N., Liska R. (2005). Photoinitiators With Functional Groups. IX. Hydrophilic Bisacylphosphine Oxides for Acidic Aqueous Formulations. InterScience.

[22] Vinicius M., Bertolo L., De Cássia R., Moraes M. (2017). Influence of Photoinitiator System on Physical-Chemical Properties of Experimental Self-Adhesive Composites. Braz. Dent. J..

[23] Chiu C.C. (2016). Liquid Bis-acylphosphine Oxide (BAPO) Photoinitiators. US Patent.

[24] Moore B.K., Platt J.A., Borges G., Chu T.M., Katsilieri I. (2008). Depth of cure of dental resin composites: ISO 4049 depth and microhardness of types of materials and shades. Oper Dent..

[25] Flury S., Hayoz S., Peutzfeldt A., Hüsler J., Lussi A. (2012). Depth of cure of resin composites: Is the ISO 4049 method suitable for bulkfill materials?. Dent. Mater..

[26] Ferracane J.L. (1985). Correlation between hardness and degree of conversion during the setting reaction of unfilled dental restorativeresins. Dent. Mater..

[27] Park Y., Chae K., Rawls H.R. (1999). Development of a new photoinitiation system for dental light-cure composite resins. Dent. Mater..

[28] Brandt W.C., de Oliveira Tomaselli L., Correr-Sobrinho L., Sinhoreti M.A.C. (2011). Can phenyl-propanedione influence Knoop hardness, rate of polymerization and bond strength of resin composite restorations?. J. Dent..

[29] Cunha Brandt W., Schneider L.F.J., Frollini E., Correr-Sobrinho L., Sinhoreti M.A.C. (2010). Effect of different photo-initiators and light curing units on degree of conversion of composites. Dent. Mater..

[30] de Resende L.F.M., Catelan A., Baroudi K., Palialol A.R.M., de Resende A.M., Andreucci A.C., Zanatta R.F., Liporoni P.C.S. (2022). Mechanical Properties of Experimental Composites with Different Photoinitiator. Eur. J. Dent..

[31] Almeida S.M., Meereis C.T.W., Leal F.B., Carvalho R.V., Boeira P.O., Chisini L.A., Cuevas-Suárez C.E., Lima G.S., Piva E. (2020). Evaluation of alternative photoinitiator systems in two-step self-etch adhesive systems. Dent. Mater..

[32] Favarao J., Oliveira D.C.R.S., Zanini M.M., Rocha M.G., Correr-Sobrinho L., Sinhoreti M.A.C. (2021). Effect of curing-light attenuation on color stability and physical and chemical properties of resin cements containing different photoinitiators. J. Mech. Behav. Biomed. Mater..

[33] Salgado V.E., Borba M.M., Cavalcante L.M., De Moraes R.R., Schneider L.F. (2015). Effect of Photoinitiator Combinations on Hardness, Depth of Cure, and Color of Model Resin Composites. J. Esthet. Restor. Dent..

[34] Price R.B., Felix C.A. (2009). Effect of delivering light in specific narrow bandwidths from 394 to 515nm on the micro-hardness of resin composites. Dent. Mater..

[35] Rocha M.G., de Oliveira D., Sinhoreti M., Roulet J.F., Correr A.B. (2019). The Combination of CQ-amine and TPO Increases the Polymerization Shrinkage Stress and Does Not Improve the Depth of Cure of Bulk-fill Composites. Oper Dent..

[36] Wang L., D’Alpino P.H., Lopes L.G., Pereira J.C. (2003). Mechanical properties of dental restorative materials: Relative contribution of laboratory tests. J. Appl. Oral. Sci..

[37] Kowalska A., Sokolowski J., Gozdek T., Kopacz K., Bociong K. (2021). The influence of various photoinitiators on the properties ofcommercial dental composite. Polymers.

[38] Oliver W.C., Pharr G.M. (1992). An improved technique for determining hardness and elastic modulus using load and displace-mentsensing indentation experiments. J. Mater. Res..

[39] (2019). Dentistry—Polymer-Based Restorative Materials.

